# Two new species of *Neozoanthus* (Cnidaria, Hexacorallia, Zoantharia) from the Pacific

**DOI:** 10.3897/zookeys.246.3886

**Published:** 2012-11-29

**Authors:** James Davis Reimer, Yuka Irei, Takuma Fujii

**Affiliations:** 1 Molecular Invertebrate Systematics and Ecology Laboratory, Rising Star Program, Trans-disciplinary Organization for Subtropical Island Studies, University; 2 of the Ryukyus, 1 Senbaru, Nishihara, Okinawa 903-0213, Japan; 3 Marine Biodiversity Research Program, Institute of Biogeosciences, Japan Agency for Marine-Earth Science and Technology (JAMSTEC), 2-15 Natsushima, Yokosuka, Kanagawa 237-0061, Japan; 4 Graduate School of Engineering and Science, University of the Ryukyus, 1 Senbaru, Nishihara, Okinawa 903-0213, Japan

**Keywords:** Zoanthid, Great Barrier Reef, Okinawa, *Neozoanthus*, new species

## Abstract

The zoanthid genus *Neozoanthus* was originally described in 1972 from a single species in Madagascar. This monotypic genus was placed within its own family, Neozoanthidae, given its unusual characters of only partial sand encrustation, and an endodermal sphincter muscle combined with a brachycnemic mesenterial arrangement. Recently, undescribed specimens of *Neozoanthus* were discovered thousands of kilometers away in both Australia and Japan. While the phylogenetic and evolutionary aspects of *Neozoanthus* spp. are now somewhat well understood, the new specimens remained undescribed. Here we describe the specimens as two new species, *Neozoanthus uchina* **sp. n.** from the Middle Ryukyu Islands of southern Japan, and *Neozoanthus caleyi* **sp. n.** from the waters around Heron Island, on the Great Barrier Reef in Australia. Both species can be distinguished from each other and the type species, *Neozoanthus tulearensis*, by their distributions, oral disk colors, and average numbers of tentacles. Additionally, each species appears to have subtle differences in their cnidae. The division of Japanese and Australian specimens into two species is strongly supported by recently reported phylogenetic data. The discovery and description of these two species highlights how little is known of zoanthid species diversity in the Indo-Pacific.

## Introduction

Zoanthids are a hexacorallian order (Zoantharia =Zoanthidea) of benthic anthozoans with gross morphological characteristics partially reminiscent of both hard corals and sea anemones. Similar to many Scleractinia, most zoanthid species are colonial, with individual polyps connected by common tissue (=coenenchyme). However, like actiniarians, most zoanthids do not secrete hard skeletons. Instead most zoanthids incorporate sand and/or detritus into their body walls to help strengthen their structure. There are, however, exceptions to these general characters within Zoantharia, with some solitary (e.g. the genus *Sphenopus*), non-encrusting (Zoanthidae; *Zoanthus*, *Acrozoanthus*, *Isaurus*), and skeleton-secreting (*Savalia*) taxa.

While zoanthids can be found in a wide variety of marine environments from shallow waters to the deep sea, much of their diversity is found in subtropical and tropical coral reef ecosystems, particularly within the suborder Brachycnemina (Swain 2010). The suborder Brachycnemina includes both encrusting (Sphenopidae) and non-encrusting (Zoanthidae) families, grouped together by possession of a mesodermal sphincter, unlike species in suborder Macrocnemina, which generally possess an endodermal sphincter (but see Swain 2010). Additionally, most coral reef brachycnemic zoanthids are in symbiosis with endosymbiotic dinoflagellate zooxanthellae (=*Symbiodinium* spp.). The most common genera are *Zoanthus* (Zoanthidae) and *Palythoa* (Sphenopidae), which are a major component of coral reef fauna in both the Atlantic and Indo-Pacific. The other coral reef zoanthid genera in Brachycnemina have been little studied due in part to their rarity or cryptic nature, and include the genera *Acrozoanthus* (Ryland 1997; Reimer et al. 2011c) and *Isaurus* (Reimer et al. 2008) in the family Zoanthidae, and the genus *Sphenopus* (Soong et al. 1999; Reimer et al. 2012) in the Sphenopidae.

There is one additional family of brachycnemic zoanthids, the monotypic Neozoanthidae. Neozoanthidae was erected by Herberts in 1972 to contain the genus and species *Neozoanthus tulearensis*, described from unusual zoanthid specimens found in coral reefs of Madagascar. The specimens were notable for zoanthids in that they had an endodermal sphincter (Herberts 1972: 139, fig. 11) but brachycnemic mesentery arrangement, unlike all other Brachycnemina. Furthermore, specimens were only partially encrusted with sand by having no encrustations around the oral ends (=tops) of polyps (Herberts 1972: 139, fig. 10). This genus has remained monotypic, and until the recent rediscovery of undescribed *Neozoanthus* specimens from the Indo-Pacific (Reimer et al. 2011a), no additional specimens had been noted in the literature.

**Figure 1.**
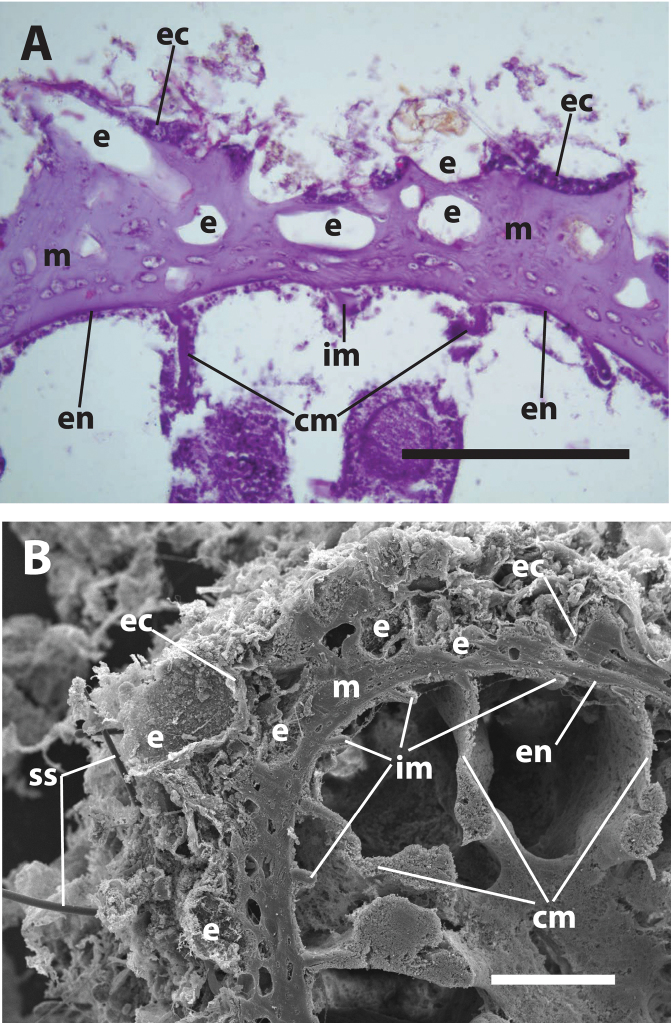
Internal structure of *Neozoanthus uchina* sp. n. showing encrustation in outer mesoglea and ectoderm, characteristic of *Neozoanthus* spp., with irregularly-sized encrustation **A** light microscope histological cross-section, and **B** scanning electron microscope image. Both images of specimen RMNH Coel 40098 (Table 2). Abbreviations: **cm**=complete mesenteries, **e**= sand/detritus encrustation (in **B**) or where encrustation existed before decalcification (in **A**), **im**=incomplete mesenteries, **ec**=ectoderm, **en**=endoderm, **m**=mesoglea, **ss**=encrusted sponge spicules. Scales: **A**=100 µm, **B**=200 µm.

*Neozoanthus* represents a unique evolutionary step in the zoanthid phylogeny as the only partially encrusted group of zoanthids (Reimer et al. 2011a) (Figure 1) (Table 1). Surprisingly, from mitochondrial 16S ribosomal DNA phylogenetic analyses, this group appears to be very closely related to the genus *Isaurus*, yet also has an indel unique to tropical macrocnemic Hydrozoanthidae (Reimer et al. 2011a), indicating *Neozoanthus* has a unique and perhaps complex evolutionary history.

In this study, utilizing both morphological and molecular techniques, we formally describe two new *Neozoanthus* species from subtropical regions of the Great Barrier Reef, Australia and the Ryukyu Archipelago, Japan by examining specimens recently reported in Reimer et al. (2011a). It is hoped these formal descriptions will provide a basis for future research into this enigmatic genus of zoanthids.

**Table 1.** Summary of morphological characters of major brachycnemic zoanthid genera compared with specimens examined in this study (adapted from Reimer et al. 2011a) to show placement of specimens within *Neozoanthus* Herberts, 1972.

**Genus**	**Encrustation?**	**Sphincter complexity**	**Sphincter position**	**Lacunae?**	**Mesogleal canals?**	**Endodermal invagination?**
*Palythoa*	Yes	Simple	Mesogleal	No	Yes	No
*Zoanthus*	No	Double	Mesogleal	Yes	Yes	No
*Isaurus*	No	Simple	Mesogleal	No	No	Yes
*Neozoanthus*	Partial	Simple	Endodermal	No	No	No
Specimens in this study	Partial	Simple	Endodermal	No	No	No

## Methods

### Sample collection

Specimens were collected as detailed in Reimer et al. (2011a) from locations in the Heron Island region of the Great Barrier Reef, Australia, and from the Ryukyu Islands in southern Japan (Table 2). In situ observations were also performed as detailed in Reimer et al. (2011a). Specimens were initially preserved in 70-99% ethanol.

**Table 2.** *Neozoanthus* specimens examined in this study with cytochrome oxidase subunit I (COI) and mitochondrial 16S ribosomal DNA (mt 16S rDNA) GenBank Accession Numbers. Data based on similar table in Reimer et al. (2011a).

**Specimen number**	**Species**	**Collection location**	**Latitude and longitude**	**Depth (m)**	**Collection date**	**Collector(s)**	**COI**	**mt 16S rDNA**
NSMT Co1554	*Neozoanthus caleyi*	North West Reef, GBR, Australia	23.3180°S, 151.7170°E	10	Nov. 17, 2009	JD Reimer	NA	NA
HI141	*Neozoanthus caleyi*	Sykes Reef, GBR, Australia	23.4322°S, 152.034°E	21	Nov. 18, 2009	JD Reimer	HM991247"	HM991230"
HI142	*Neozoanthus caleyi*	Sykes Reef, GBR, Australia	23.4322°S, 152.0338°E	21	Nov. 18, 2009	JD Reimer	HM991248"	HM991231"
HI143	*Neozoanthus caleyi*	Sykes Reef, GBR, Australia	23.4322°S, 152.0338°E	21	Nov. 18, 2009	JD Reimer	NA	HM991232"
HI144	*Neozoanthus caleyi*	Sykes Reef, GBR, Australia	23.4322°S, 152.0338°E	20	Nov. 18, 2009	JD Reimer	NA	HM991233"
HI145	*Neozoanthus caleyi*	Sykes Reef, GBR, Australia	23.4322°S, 152.0338°E	18	Nov. 18, 2009	JD Reimer	HM991249"	HM991234"
HI199	*Neozoanthus caleyi*	Heron Channel, GBR, Australia	23.4448°S, 151.9504°E	22	Nov. 22, 2009	JD Reimer	NA	NA
HI200	*Neozoanthus caleyi*	Heron Channel, GBR, Australia	23.4448°S, 151.9504°E	23	Nov. 22, 2009	JD Reimer	HM991250"	HM991235"
HI209	*Neozoanthus caleyi*	Sykes Reef, GBR, Australia	23.4322°S, 152.0338°E	28	Nov. 23, 2009	JD Reimer	HM991251"	HM991236"
HI214	*Neozoanthus caleyi*	Sykes Reef, GBR, Australia	23.4322°S, 152.0338°E	9	Nov. 23, 2009	JD Reimer	HM991252"	HM991237"
MTQ G65793	*Neozoanthus caleyi*	Sykes Reef, GBR, Australia	23.4322°S, 152.0338°E	4	Nov. 23, 2009	JD Reimer	HM991253"	HM991238"
HI224	*Neozoanthus caleyi*	Heron Channel, GBR, Australia	23.4532°S, 151.9005°E	26	Nov. 24, 2009	JD Reimer	HM991254"	HM991239"
HI225	*Neozoanthus caleyi*	Heron Channel, GBR, Australia	23.4532°S, 151.9005°E	25	Nov. 24, 2009	JD Reimer	HM991255"	HM991240"
HI227	*Neozoanthus caleyi*	Heron Channel, GBR, Australia	23.4532°S, 151.9005°E	25	Nov. 24, 2009	JD Reimer	HM991256"	HM991241"
HI231	*Neozoanthus caleyi*	Heron Channel, GBR, Australia	23.4530°S, 151.9171°E	23	Nov. 24, 2009	JD Reimer	HM991257"	HM991242"
HI101114-13	*Neozoanthus caleyi*	Sykes Reef, GBR, Australia	23.4316°S, 152.0493°E	29	Nov. 14, 2010	JD Reimer	NA	NA
RMNH Coel 40098	*Neozoanthus uchina*	Manza, Okinawa, Japan	26.5047°N, 127.8450°E	25	Sept. 1, 2008	JD Reimer et al.	NA	NA
MISE 545	*Neozoanthus uchina*	Teniya, Okinawa, Japan	26.5638°N, 128.1408°E	1 to 2	Sept. 5, 2008	JD Reimer et al.	NA	NA
MISE 546	*Neozoanthus uchina*	Teniya, Okinawa, Japan	26.5638°N, 128.1408°E	1 to 2	Sept. 5, 2008	JD Reimer et al.	NA	NA
USNM 1194728	*Neozoanthus uchina*	Teniya, Okinawa, Japan	26.5638°N, 128.1408°E	1 to 2	Sept. 5, 2008	JD Reimer et al.	HM991246"	HM991227"
MISE 549	*Neozoanthus uchina*	Teniya, Okinawa, Japan	26.5638°N, 128.1408°E	1 to 2	Sept. 5, 2008	JD Reimer et al.	NA	NA
NSMT Co1553	*Neozoanthus uchina*	Teniya, Okinawa, Japan	26.5638°N, 128.1408°E	1 to 2	Sept. 5, 2008	JD Reimer et al.	HM991243"	NA
MISE 560	*Neozoanthus uchina*	Yona, Okinawa, Japan	26.7684°N, 128.1976°E	13	Sept. 24, 2008	JD Reimer, T Fujii	NA	NA
MISE 1092	*Neozoanthus uchina*	Teniya, Okinawa, Japan	26.5638°N, 128.1408°E	1	July 2008	JD Reimer	NA	NA
MISE 1093	*Neozoanthus uchina*	Teniya, Okinawa, Japan	26.5638°N, 128.1408°E	Inter-tidal	July 2008	JD Reimer	NA	NA
MISE 1115	*Neozoanthus uchina*	Tinyuhama, Korijima, Okinawa, Japan	26.7149°N, 128.0127°E	24	Dec. 28, 2008	JD Reimer	HM991245"	HM991228"
MISE 1116	*Neozoanthus uchina*	Tinyuhama, Korijima, Okinawa, Japan	26.7149°N, 128.0127°E	24	Dec. 28, 2008	JD Reimer	HM991244"	HM991229"
MISE 1400	*Neozoanthus uchina*	Omonawa, Tokunoshima, Kagoshima, Japan	27.6669°N, 128.9685°E	9	March 9, 2010	JD Reimer	NA	NA
MISE 1401	*Neozoanthus uchina*	San, Tokunoshima, Kagoshima, Japan	27.8693°N, 128.9699°E	10	March 10, 2010	JD Reimer	NA	NA
MISE 1402	*Neozoanthus uchina*	San, Tokunoshima, Kagoshima, Japan	27.8693°N, 128.9699°E	12	March 10, 2010	JD Reimer	NA	NA
MISE 1403	*Neozoanthus uchina*	Zampa, Okinawa, Japan	26.4414°N, 127.7119°E	NA	August 29, 2008	JD Reimer	NA	NA
MISE MO-100	*Neozoanthus uchina*	Tebiro, Amami-Oshima, Kagoshima, Japan	28.4013°N, 129.6178°E	10	March 16, 2011	M Obuchi	NA	NA

Abbreviations: GBR=Great Barrier Reef; NA=not acquired. Sample number abbreviations as in Methods.

### Specimen examination/decalcification/histology

Specimens were examined, decalcified, and sectioned as detailed in Reimer et al. (2011a), with additional analyses as detailed below. As detailed in Reimer et al. (2011a), two polyps from two specimens each (Australia and Japan) were examined (total n=8).

### Morphological analyses

External morphology of specimens was examined using both preserved specimens and in situ images. Polyp dimensions (oral disk diameter, polyp height) for both in situ and preserved specimens were obtained, as were the following data: tentacle number, color of polyp, color(s) of oral disk, relative amount of sand encrustation, associated/substrate species. Additionally, the relative development of the coenenchyme was examined.

For internal examinations, the following data were obtained: mesentery form (brachycnemic or macrocnemic arrangement), mesentery numbers, presence/absence of encrustations, location of encrustations, location and development of the sphincter muscle, presence/absence of gonads. Decalcification, histology and electron microscopy were performed as described in Reimer et al. (2011a).

### Nematocyst observation

Undischarged nematocysts were measured from tentacles, column, actinopharynx, and mesenterial filaments of polyps (specimens examined n=2-4 colonies/species) for both new species. 400x images of the nematocysts were obtained by optical microscope, and measured using the software ImageJ (National Institutes of Health, USA). Nematocyst nomenclature generally followed England (1991), however both Schmidt (1974) and Hidaka and co-workers (1987; 1992) have previously suggested basitrichs and mastigophores are same type of the nematocyst, and thus in this study, these two types were dealt with as the same type (basitrichs and *b*-mastigophores), unless they could be clearly distinguished from one another (basitrichs and *p*-mastigophores), in which case they were analyzed separately. Both holotypes and all paratypes of both newly described species were examined.

### DNA extraction and PCR amplification/Phylogenetic analyses

Phylogenetic analyses are detailed and were performed on both species in Reimer et al. (2011a). No new genetic analyses were performed in this study. DNA from specimens were extracted, DNA target regions (cytochrome oxidase subunit I; mitochondrial 16S ribosomal DNA) amplified by PCR, and sequences analysed as detailed in Reimer et al. (2011a). DNA alignments from Reimer et al. (2011a) are available from the corresponding author.

### Abbreviations used

MTQMuseum of Tropical Queensland, Townsville, Australia.


USNMSmithsonian National Museum of History, Washington D.C., USA


NSMTNational Museum of Nature and Science, Tokyo, Japan


RMNHNaturalis Biodiversity Center, Leiden, the Netherlands


MISEMolecular Invertebrate Systematics and Ecology Laboratory, University of the Ryukyus, Nishihara, Okinawa, Japan


## Systematics

Additional data related to both species, including tables, phylogenetic trees, and histological images, are reported in Reimer et al. (2011a). The specimens in Reimer et al. (2011a) were placed into the genus *Neozoanthus* Herberts, 1972 based on the summary of morphological characters (Table 1).

### Family Neozoanthidae Herberts, 1972

**Figure 2.**
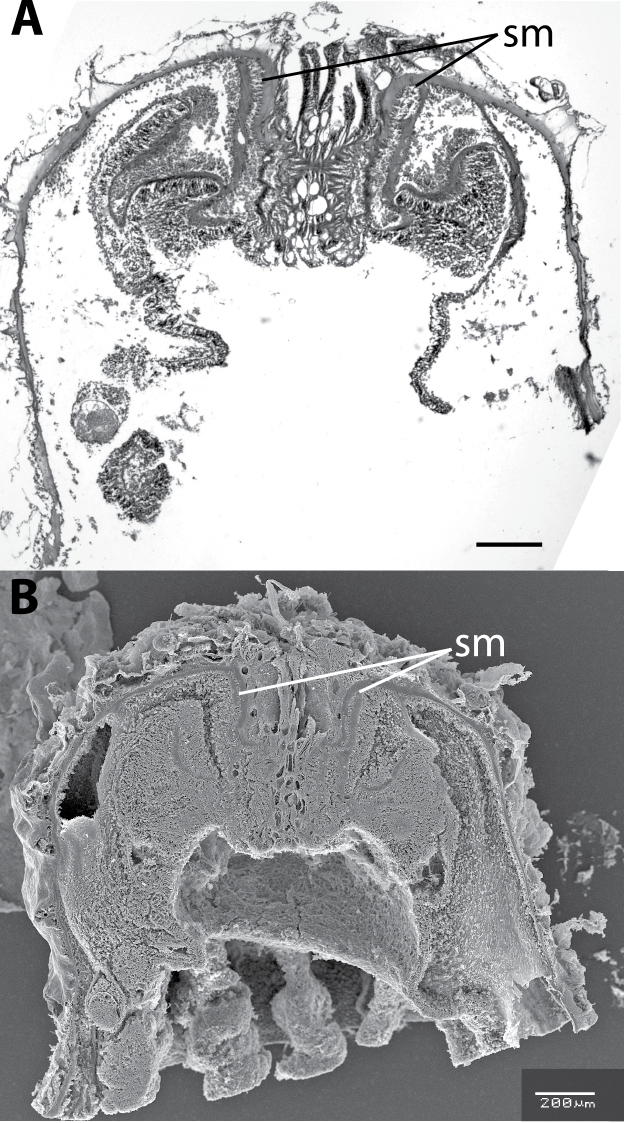
Longitudinal section of *Neozoanthus caleyi* sp. n. specimen HI225 showing endodermal sphincter muscle (=sm). **A** Light microscope **B** scanning electron microscope. Both scales =200 µm.

**Diagnosis.**Brachycnemic zoanthids with a simple endodermal sphincter muscle (Figure 2) that are only partially sand-encrusted (Figure 1).

### Genus *Neozoanthus* Herberts, 1972

**Type species.** *Neozoanthus tulearensis* Herberts, 1972

**Diagnosis.**As for the family above.

#### Neozoanthus caleyi sp. n.

urn:lsid:zoobank.org:act:3BD527A8-F3CC-4A09-A933-313EBAE6A45C

http://species-id.net/wiki/Neozoanthus_caleyi

Figures 2 3 5A Tables 2 3 S1

Neozoanthus caleyi Synonymy: “GBR clade” of *Neozoanthus*Reimer et al. 2011a: fig. 2.
Neozoanthus caleyi *“Neozoanthus* sp. Australia” *-*Reimer et al. 2011a: 986, 989, fig. 4.

##### Material examined.

**Type specimens.** Holotype, specimen number MTQ G65793. Colony in two pieces, 5 polyps on a 2.5 × 2.0 cm stone and 4 polyps on a 2.0 × 1.0 cm stone (originally one colony). Polyps approximately 2.3–5.0 mm in diameter, and approximately 2.5–3.0 mm in height from stoloniferous coenenchyme. Polyps and coenenchyme encrusted with irregularly sized and colored sand grains. There was no noticeable variation between holotype and other specimens. Preserved in 99.5% ethanol.

*Paratype* (fromAustralia): Paratype 1. Specimen number NSMT Co1554. North West Reef, Queensland, at 10 m by JDR, November 17, 2009.

**Type locality.**Australia, Queensland: Great Barrier Reef, Sykes Reef, 23.4322°S, 152.0338°E, reef with coral rubble, at 4 m, 23 November 2009, JDR leg.

**Other material** (all from Great Barrier Reef, Queensland, Australia; coll. JDR): Sykes Reef MISE HI-141 to 145 (n=5), 18-21 m, 18 November 2009; MISE HI-209, 28 m, 23 November 2009; MISE HI-214, 9 m, 23 November 2009; MISE HI101114-13, 29 m, 14 November 2010. Heron Island Channel, MISE HI-199 to 200 (n=2), 22-23 m, 22 November 2009; MISE HI-224 to 225, 227 (n=3), 25-26 m, 24 November 24 2009; MISE HI-231, 23 m, 24 November 2009 (see also Table 2).

##### Description.

*Size*: Polyps in situ approximately 2–5 mm in diameter when open, and approximately 2–3 mm in height.

**Figure 3.**
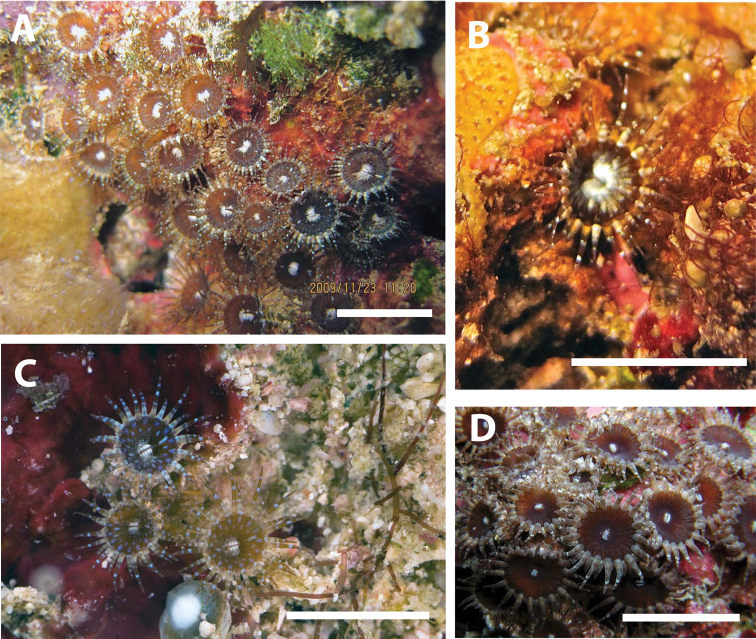
*Neozoanthus caleyi* sp. n. in situ around Heron Island on the Great Barrier Reef, Queensland, Australia. **A** Specimen HI214 at Sykes Reef, depth=9 m, November 23, 2009 **B** Close-up of a single polyp showing yellow coloration at base of tentacles; specimen HI145 at Sykes Reef, depth=18 m, November 18, 2009 **C** Specimen HI231 at Heron Channel, depth=23 m, November 24, 2009 **D** Uncollected specimen at Heron Channel, depth=approximately 20 m, November 2011. Scales approximately 1 cm. **A, B** taken by JD Reimer, **C, D** taken by Gary Cranitch.

*Morphology:* *Neozoanthus caleyi* sp. n.has 28 to 40 (average 33±3.9, n=18 polyps on 8 colonies) conical tentacles. Tentacles are usually shorter than the expanded oral disk diameter (e.g. 50-80% of oral disk width). Tentacles may be grayish-blue, yellow, or transparent, often with black, white, or fluorescent blue bands or patterning (Figure 3). Well-developed, simple endodermal sphincter. No bractae are visible. All specimens are zooxanthellate. Polyps are externally heavily encrusted with sand and other particles of irregular sizes, excepting the oral end, which is free of encrustation and appears a bluish-gray similar to as seen in some *Zoanthus* species. When fully contracted, the sand-free oral end is often not visible, and polyps resemble small balls of sand. Polyps extend well clear of reduced or stoloniferous coenenchyme (Figure 3). Oral disks may be a variety of colors, including light gray-blue, white, or deep wine red. Occasionally, white, yellow, or light blue dots may be seen on the oral disk in regular circular patterns, and the oral opening (mouth) is often white in color. A “skirt” of different coloration (usually white or lighter coloration than remainder of oral disk) covering up to approximately 90 degrees of the oral disk is often seen in the area of the dorsal directive. Colonies consist of tens to <100 polyps, connected by stolons with no well-developed coenenchyme.

*Cnidae*: Basitrichs and microbases (often difficult to distinguish), holotrichs (large and small), spirocysts (see Table S1, Figure 5).

##### Differential diagnosis.

Differs from *Neozoanthus tulearensis* Herberts, 1972 and *Neozoanthus uchina*sp. n. with regards to distribution (southern Great Barrier Reef as opposed to Madagascar and Ryukyu Archipelago, respectively), coloration (no yellow observed in any *Neozoanthus uchina* sp. n.), and tentacle count (*Neozoanthus tulearensis* = 38 to 44 tentacles (n= 8 colonies; 18 polyps), *Neozoanthus uchina* sp. n. = average 38±3.0 tentacles, n= 9 colonies; 24 polyps). The two new *Neozoanthus* species’ tentacle counts are statistically significant (t-test, p<0.001). The two new *Neozoanthus* species mt 16S rDNA sequences differ by three base pairs (Reimer et al. 2011a).

##### Etymology.

Named for Dr. Julian Caley, the leader of the Australian Census of Coral Reef Ecosystems (CReefs) project. Dr. Caley’s acceptance of the first author’s participation in CReefs led to the discovery of this species. Noun in genitive.

##### Habitat, ecology and distribution.

Specimens from the Great Barrier Reef were found at depths from 4 to 29 m. Despite repeated surveys, no *Neozoanthus caleyi* sp. n. have been found further north around Lizard Island despite zoanthid-focused surveys (Burnett et al. 1997; J.D. Reimer & T. Fujii, unpublished data), and it may be that this species is limited to a subtropical distribution in the Great Barrier Reef.

*Neozoanthus caleyi* sp. n., although not found at many locations surveyed, was locally common, particularly at locations that were characterized by strong currents and some sedimentation, with large coarse sand particles scattered over the bottom or rocks, for example on the bottom of Heron Channel. Preference for such environments may be related to its encrustation patterns. Colonies were never found in locations completely exposed to light, yet all colonies were zooxanthellate. Most colonies were relatively small, consisting of tens (not hundreds) of polyps, with polyps spread out and connected by thin stolons (Figure 3).

##### Notes.

This species can close its polyps much more rapidly than those of other zooxanthellate zoanthid genera (Reimer pers. obs).

**Table 3.** Comparison of various features of *Neozoanthus tulearensis* Herberts, 1972, *Neozoanthus caleyi* sp.n. and *Neozoanthus uchina* sp. n.

**Morphological character**	***Neozoanthus tulearensis***	***Neozoanthus uchina* sp. n**.	***Neozoanthus caleyi* sp. n**.
Distribution	NE Madagascar	Middle Ryukyu Islands, Okinawa, Japan	Heron Island, Great Barrier Reef, Australia
Depth	No data	Intertidal to 25 m	4 to 29 m
Oral disk color	Greenish-beige to yellow	Light gray-blue, white, rust or deep wine red	Light gray-blue, white, or deep wine red
Polyp diameter (mm)	1.5 to 5.0	2.2 to 5.1	2.3 to 5.0
Polyp height (mm)	2.0 to 12.0	2.0 to 8.5	2.5 to 3.0
Number of tentacles (avg. ± SE)	38–44	32–42 (38±3.0)	28–40 (33±3.9)
Cnidae			
Column	Microbasic mastigophores	Holotrichs	Holotrichs
Pharynx	Microbasic mastigophores	Holotrichs, basitrichs, spirocysts	Holotrichs, basitrichs
Tentacles	Holotrichs, spirocysts	Holotrichs, basitrichs, spirocysts	Holotrichs, basitrichs, spirocysts
Filaments	Holotrichs, microbasic mastigophores	Holotrichs, *p*-mastigophores	Holotrichs, basitrichs, *p*-mastigophores

##### DNA Sequences.

Originally listed in Table S1 in Reimer et al. (2011a).

Cytochrome oxidase subunit I: HM991247-HM991257

Mitochondrial 16S ribosomal DNA: HM991230-HM991242

#### Neozoanthus uchina sp. n.

urn:lsid:zoobank.org:act:BBAB21D0-275E-4287-90F4-4928CE1BF05E

http://species-id.net/wiki/Neozoanthus_uchina

Figures 1 4 5B Tables 2 3 S1

**Figure 4.**
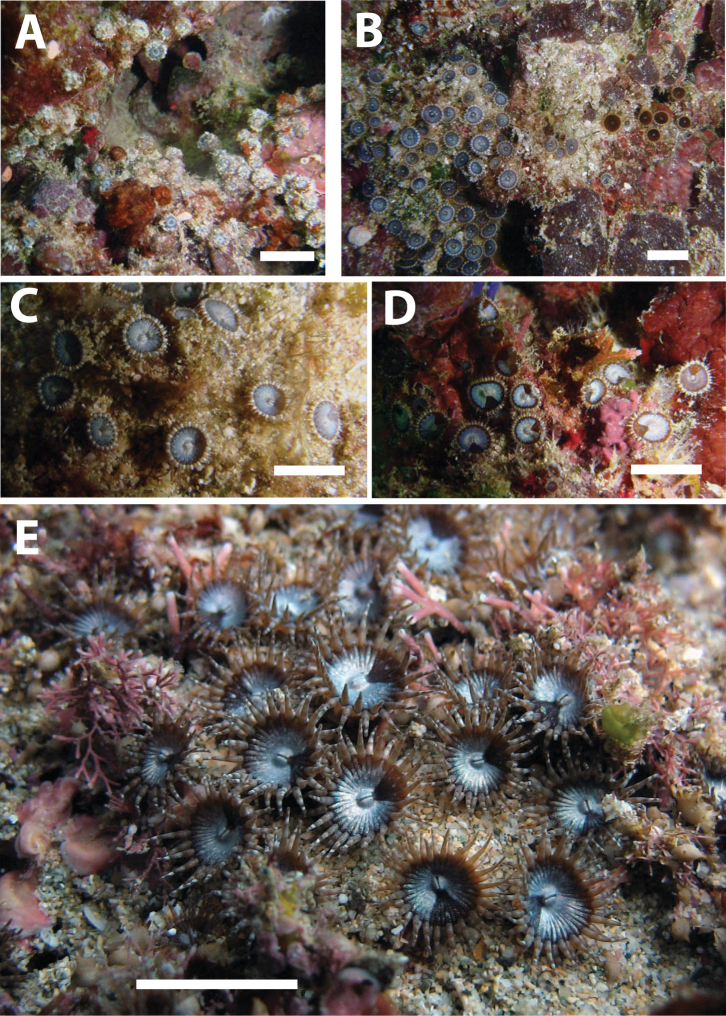
*Neozoanthus uchina* sp. n. in situ. **A** Partially closed polyps showing lack of encrustation at oral end **B** Colonies of two different color morphotypes **C** Close-up of polyps of the same color morphotype as on the left in **B**) **D** Polyps showing variation in oral disk color where the dorsal directive is located. Scales approximately 1 cm. **A** to **D** images taken by Masaru Mizuyama, September 20, 2010, in the lower intertidal zone at Kamomine, Tokunoshima, Kagoshima, Japan, specimens uncollected **E** Colony MISE MO-100 in situ on March 16, 2011 at Tebiro Beach, Amami-oshima, Kagoshima, Japan. Image taken by Masami Obuchi.

Neozoanthus uchina Synonymy: “*Neozoanthus* sp. okinawa” – Reimer 2010: 25, 27, fig. 8.
Neozoanthus uchina : Reimer et al. 2011a: 986, 989, fig. 4.
Neozoanthus uchina : “Japan clade” of *Neozoanthus* - Reimer et al. 2011a: fig. 2.

##### Material examined.

##### Type specimens.

Holotype, specimen number NSMT-Co1553. Colony of 17 polyps connected by stoloniferous coenenchyme on a rock approximately 4.5 × 3.0 cm. Polyps approximately 2.0–4.4 mm in diameter, and approximately 2.0–5.4 mm in height from coenenchyme. Polyps and coenenchyme encrusted with irregularly sized and colored sand grains. There was no noticeable variation between holotype and other specimens. Preserved in 99.5% ethanol. Original label.

*Paratypes* (all from Japan): Paratype 1. Specimen number USNM 1194728. Collected from Teniya, Nago, Okinawa, at 1 to 2 m by JDR, September 5, 2008. Paratype 2. Specimen number RMNH Coel 40098. Collected from Manza, Onna, Okinawa I., Japan, at 25 m by JDR, 1 September, 2008.

**Type locality**. Japan, Okinawa Prefecture, Okinawa Island: Nago City, Teniya, 26.563832°N, 128.140822°E, in small cracks on reef flat at 1 to 2 m depth, 5 September 2008, J.D. Reimer (JDR) leg.

**Other material** (all from Japan, coll. JDR unless noted): Teniya, Okinawa I., Okinawa, MISE 545, 546, 549 (n=3), 1-2 m 5 September 2008; Yona, Okinawa I., Okinawa, MISE 560, 13 m, coll. JDR and Takuma Fujii (TF), 24 September 2008; Teniya, Okinawa I., Okinawa, MISE 1092, 1093 (n=2), intertidal - 1 m, 1 July 2008; Tinyuhama, Korijima I., Okinawa, MISE 1115, 1116 (n=2), 24 m, 28 December 2008; Omonawa, Tokunoshima I., Kagoshima, MISE 1400, 9 m, 9 March 2010; San, Tokunoshima I., Kagoshima, MISE 1401, 1402 (n=2), 10-12 m, 10 March 2010; Zampa, Okinawa I., Okinawa, MISE 1403, at unknown depth, 29 August 2008 (see also Table 2); Tebiro Beach, Amami-Oshima I., Kagoshima, MISE MO-100, 10 m, coll. Masami Obuchi, 16 March 2011.

##### Description.

*Size*: Polyps in situ approximately 2.2-5.1 mm in diameter when open, and approximately 2-8.5 mm in height.

*Morphology:* *Neozoanthus uchina* sp. n.has 32 to 42 (average 38±3.0, n=24 polyps on 9 colonies) conical tentacles. Tentacles are usually shorter than the expanded oral disk diameter (e.g. 50-80% of oral disk width). Tentacles may be grayish-blue, rust red, or transparent, often with black, white, or fluorescent blue bands or patterning (Figure 4). No bractae are visible, and all specimens were zooxanthellate. Polyps are externally heavily encrusted with sand and other particles of irregular sizes, excepting the oral end, which is free of encrustation and appears a bluish-gray similar to as seen in some *Zoanthus* species. When fully contracted, the sand free oral end is often not visible, and polyps resemble small balls of sand. Polyps extend well clear of reduced or stoloniferous coenenchyme (Figure 4). Oral disks may be a variety of colors, such as light gray-blue, white, rust or deep wine red. Occasionally, white or light blue dots may be seen on the oral disk in regular circular patterns, and the oral opening (mouth) is often white or cream in color. A “skirt” of different coloration (usually white or lighter coloration than remainder of oral disk) covering up to approximately 90 degrees of the oral disk is often seen in the area of the dorsal directive. Colonies consisted of tens to <100 polyps, connected by stolons with no well-developed coenenchyme.

**Figure 5.**
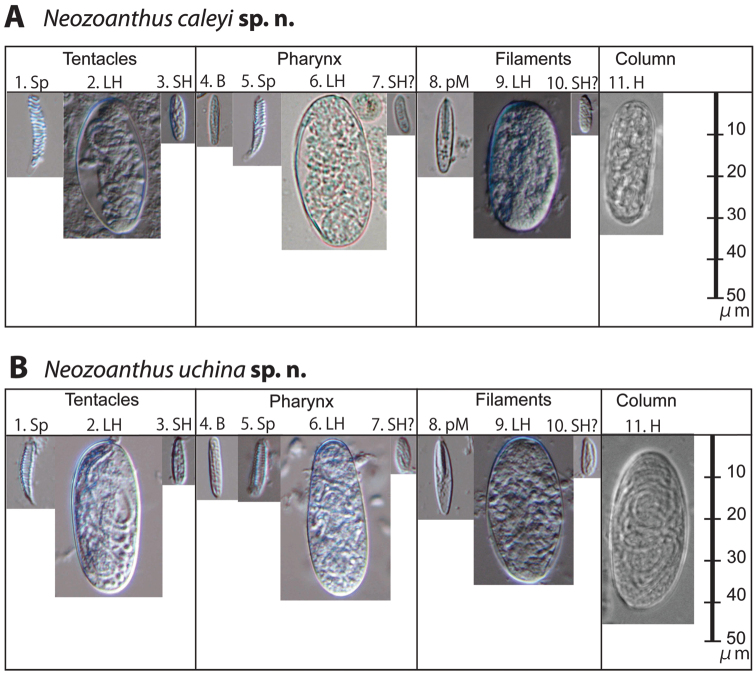
Cnidae of *Neozoanthus caleyi* sp. n. and *Neozoanthus uchina* sp. n. from the tentacles, pharynx, and filaments showing their relative size. Type abbreviations: **Sp**=spirocysts, **H**=holotrichs, **LH**=large holotrichs, **SH**=small holotrichs, **B**=basitrichs, **SH?**=potential small holotrichs, **pM**=*p*-mastigophores. Size and frequency data are given in Table S1.

*Cnidae*: Basitrichs and microbasic *p*-mastigophores (often difficult to distinguish), holotrichs (large and small), spirocysts (see Table S1, Figure 5).

##### Differential diagnosis.

Differs from *Neozoanthus tulearensis* Herberts, 1972 and *Neozoanthus caleyi* sp. n. with regards to distribution (Ryukyu Archipelago as opposed to Madagascar and southern Great Barrier Reef, respectively), coloration (yellow observed in some *Neozoanthus caleyi* sp. n.), and tentacle count (*Neozoanthus tulearensis* = 38 to 44 tentacles, *Neozoanthus caleyi* sp. n. = average 33±3.9 tentacles). The two new *Neozoanthus* species’ tentacle counts are statistically significant (t-test, p<0.001). Often polyps are much taller (to 8.5 mm) than *Neozoanthus caleyi* sp. n. (to 3.0 mm), although height ranges overlap (Table 3). The two new *Neozoanthus* species mt 16S rDNA sequences differ by three base pairs (Reimer et al. 2011a).

*Neozoanthus uchina* sp. n.is currently the only partially encrusted zoanthid described from the Ryukyu Archipelago.

##### Etymology.

Named for the Okinawan dialect word for Okinawa, “uchina”, the prefecture where this species was first found. Noun in apposition.

##### Habitat, ecology, and distribution.

Specimens from the Ryukyu Archipelago were found at depths from the intertidal zone to 25 m. Despite repeated surveys focused on zoanthids, no *Neozoanthus uchina* sp. n.have been found further north on Yakushima Island or mainland Japan, nor further south in the Miyako and Yaeyama Islands of southern Okinawa, and it may be that this species is limited to a subtropical distribution in the Middle Ryukyu Islands. Additionally, despite surveys, thus far no specimens have been reported from neighboring Taiwan (Reimer et al. 2011d) or the Ogasawara Islands (Reimer et al. 2011b).

*Neozoanthus uchina* sp. n., although not found at many locations surveyed, was locally common, particularly at locations that were characterized by strong currents and some sedimentation, with large coarse sand particles scattered over the bottom or rocks. Preference for such environments may be related to its encrustation patterns. Colonies were almost always found in cracks and holes in rocks partially exposed to light, and usually not in locations completely exposed to light. Most colonies were relatively small, consisting of tens (not hundreds) of polyps, with polyps spread out and connected by thin stolons (Figure 4).

##### Notes.

This species can close its polyps much more rapidly than species of other zooxanthellate zoanthid genera (Reimer pers. obs).

##### DNA Sequences.

Originally listed in Table S1 in Reimer et al. (2011a).

Cytochrome oxidase subunit I: HM991243-HM991246

Mitochondrial 16S ribosomal DNA: HM991227-HM991229

## Discussion

### Neozoanthidae a valid family?

As stated previously (Reimer et al. 2011a), given *Neozoanthus’* close phylogenetic affiliation with *Isaurus*, a genus of the family Zoanthidae, it is unlikely that Neozoanthidae is a valid family grouping. However, given the unique morphological characters of *Neozoanthus*, as well as a unique mt 16S rDNA indel shared with Hydrozoanthidae, more molecular data from other markers are needed before Neozoanthidae is formally merged into Zoanthidae. These results clearly indicate that at least one of the diagnostic characters for erecting Neozoanthidae by Herberts (1972), sphincter muscle position, does not have utility, as the family was erected based in a large part on an endodermal (macrocnemic) sphincter muscle. As shown by Swain (2010), sphincter muscle position is apparently not diagnostic for higher level (genus, family, suborder) taxonomy in zoanthids, and furthermore, as mentioned in Reimer et al. (2011a), many morphological features in zoanthids, including the presence or absence of sand encrustation and sphincter muscle position can evolve or change relatively rapidly.

### Ecology of *Neozoanthus*

Both new species in this study were found in areas notable for their strong currents. Both species, although zooxanthellate, were found in areas somewhat sheltered from direct sunlight, unlike many *Zoanthus* and *Palythoa* spp. The sand encrustation plus *Neozoanthus* species’ preference for cracks and overhangs may have led to their lack of discovery in both Australia and Japan until 2008-2009 (Reimer et al. 2011a). Certainly, the species are not “cryptic” in the classic sense, as they have colorful oral disk patterns, but the fact that they had been overlooked until recently demonstrates the lack of attention paid to zoanthids in field surveys.

*Neozoanthus caleyi* sp. n. possesses *Symbiodinium* (=zooxanthellae) of subclade C1 *sensu* LaJeunesse (2002) (Reimer and Irei unpubl. data), a “generalist” type of *Symbiodinium* known to be sensitive to thermal stress, and the distribution patterns of *Neozoanthus uchina* sp. n. and *Neozoanthus caleyi* sp. n. fit well with this symbiont’s physiology, as both species were usually not found at depths of <10 m exposed directly to sunlight and colonies in shallower waters were in cracks or crevices that provided shading. Despite the fact that no studies have yet been conducted on their ecology, the presence of both species from shallow to deeper waters in areas of strong currents combined with somewhat long tentacles (*e.g*. compared to *Palythoa heliodiscus* with tentacles only 10% length of oral disk – Ryland and Lancaster 2003) indicates both *Neozoanthus* species may be mixotrophic, obtaining energy from both prey capture and photosymbiotic *Symbiodinium*.

As mentioned in the species’ descriptions, for now it appears that both *Neozoanthus uchina* sp. n. and *Neozoanthus caleyi* sp. n. have subtropical distributions, as no colonies were found to regions directly north (both species) or directly south (*Neozoanthus uchina* sp. n.) of their distribution. However, due to their small size and preference for semi-cryptic microhabitats, we cannot discount the possibility that there are further populations of both species that await discovery. Furthermore, it is known there are unidentified *Neozoanthus* species in Indonesia (Reimer & Hoeksema, unpublished data), and specimens are needed to complete work on these.

## Conclusions

Two new species of *Neozoanthus* from the Pacific are formally described, one from the Great Barrier Reef and one from the Middle Ryukyu Islands.

The discovery of these two species (detailed in Reimer et al. 2011a) and their relative commonness at some sampling sites indicates that much work remains to be performed in order to properly understand zoanthid diversity in the Indo-Pacific.

We recommend the utilization of the combination of both molecular results (Reimer et al. 2011a) with the morphological descriptions given in this study for zoanthid identification and description, as seen in many recent studies.

## Supplementary Material

XML Treatment for Neozoanthus caleyi

XML Treatment for Neozoanthus uchina

## Appendix

Types, relative abundance and sizes of cnidae in *Neozoanthus uchina* sp. n. and *Neozoanthus caleyi* sp. n. (doi: 10.3886/zookeys.246.3886.app) File format: Microsoft Word Document (doc).
